# Continual Trials Spontaneous Recognition Tasks in Mice: Reducing Animal Numbers and Improving Our Understanding of the Mechanisms Underlying Memory

**DOI:** 10.3389/fnbeh.2018.00214

**Published:** 2018-09-13

**Authors:** Michele Chan, Madeline J. Eacott, David J. Sanderson, Jianfei Wang, Mu Sun, Alexander Easton

**Affiliations:** ^1^Department of Psychology, Durham University, Durham, United Kingdom; ^2^Centre for Learning and Memory Processes, Durham University, Durham, United Kingdom; ^3^Department of Psychology, University of Essex, Colchester, United Kingdom; ^4^Integrated Biological Platform Sciences, Glaxo Smith Kline (GSK) R&D Co., Zhangjiang Hi-Tech Park, Shanghai, China

**Keywords:** recognition memory, proactive interference, mouse, spontaneous recognition memory, object recognition

## Abstract

Spontaneous recognition tasks are widely used as a laboratory measure of memory in animals but give rise to high levels of behavioral noise leading to a lack of reliability. Previous work has shown that a modification of the procedure to allow continual trials testing (in which many trials are run concurrently in a single session) decreases behavioral noise and thus significantly reduces the numbers of rats required to retain statistical power. Here, we demonstrate for the first time that this improved method of testing extends to mice, increasing the overall power of the approach. Moreover, our results show that the new continual trials approach provides the additional benefits of heightened sensitivity and thus provides greater insight into the mechanisms at play. Standard (c57) and transgenic Alzheimer model (TASTPM) mice were tested both at 7 and 10 months of age in both object recognition (OR) and object-location (OL) spontaneous recognition tasks using the continual trials methodology. Both c57 and TASTPM mice showed age-dependent changes in performance in OR. While c57 mice also showed age-related changes in performance of OL, TASTPM mice were unable to perform OL at either age. Significantly, we demonstrate that differences in OL performance in c57s and TASTPM animals is a result of proactive interference rather than an absolute inability to recognize OL combinations. We argue that these continual trials approaches provide overall improved reliability and better interpretation of the memory ability of mice, as well as providing a significant reduction in overall animal use.

## Introduction

Spontaneous recognition tasks have been widely used to assess memory in recent years. In these tasks, animals express their memory through spontaneous exploration of objects. Where one object has been presented in a sample phase, and a copy of that object and a novel object are presented in a test phase, animals preferentially explore the novel object (Ennaceur and Delacour, 1988). This object preference is due to the animals’ preference to explore novel objects and is an expression of memory as the novel object will only be preferentially explored if the animal is able to remember having seen the familiar object in the sample phase. Variations in this task can be used to assess memory for other aspects of the sample phase including memory for locations (Ennaceur and Meliani, 1992), object-location (OL; Dix and Aggleton, 1999) or object-context (Dix and Aggleton, 1999) configurations and even episodic memory (Eacott and Norman, 2004; Eacott et al., 2005). Being able to assess this wide variety of memory types which have been shown to be dissociable in terms of the underlying memory mechanisms (Eacott and Gaffan, 2005; Easton and Eacott, 2010) using spontaneous behavior has made the use of these tasks extremely valuable and widespread.

Although spontaneous recognition tasks have significant advantages, there are also non-trivial disadvantages to the widespread use of these tasks. Being reliant on spontaneous behavior, performance on the task can be affected by a range of factors including individual object preferences, stress (which can be exacerbated by the significant handling within a trial), external noise etc., (Ameen-Ali et al., 2015). This high level of variability leads to high levels of behavioral noise which results either in the use of large numbers of animals to produce sufficient statistical power or ambiguous results which can be misinterpreted (Ennaceur, 2010; Ameen-Ali et al., 2015). As reproducibility in neuroscience and psychology becomes an increasingly important issue alongside the poor translation of basic neuroscience research to the clinic, improvement in the reliability and interpretability of these spontaneous recognition tasks would be of significant value (Ameen-Ali et al., 2015).

In recent years, we have demonstrated a novel approach to spontaneous recognition tasks in rats that have improved behavioral reliability and thus allowed reduced use of animals (Ameen-Ali et al., 2012; Seel et al., 2017). This continual trials approach both increases the number of trials completed by an animal within a single session, and by removing within-session handling of animals, minimizes handling induced stress. Not only do these tasks offer a way of reducing behavioral variability and therefore improve reliability of the results, they also offer new windows into the mechanisms involved in these tasks, for example allowing the study of interference in these tasks over multiple consecutive trials (Seel et al., 2017).

Although the value of these new approaches have been demonstrated in rats (Ameen-Ali et al., 2012; Seel et al., 2017), increasing use of transgenic mouse models of disease suggest that there is significant benefit in additionally applying these new approaches in mice. Here, we show that an adapted version of the continual trials apparatus can be used successfully in groups of c57 and (later) in transgenic TASTPM mice. These mice were chosen in order to demonstrate use of the procedure to examine normal performance and its sensitivity to detect changes in performance in a memory-impaired strain. In addition, we demonstrate that the continual trials approach allows clearer understanding of the processes involved in recognition memory, such as the nature of proactive interference.

## Materials and Methods

### Apparatus

**Figure 1:**
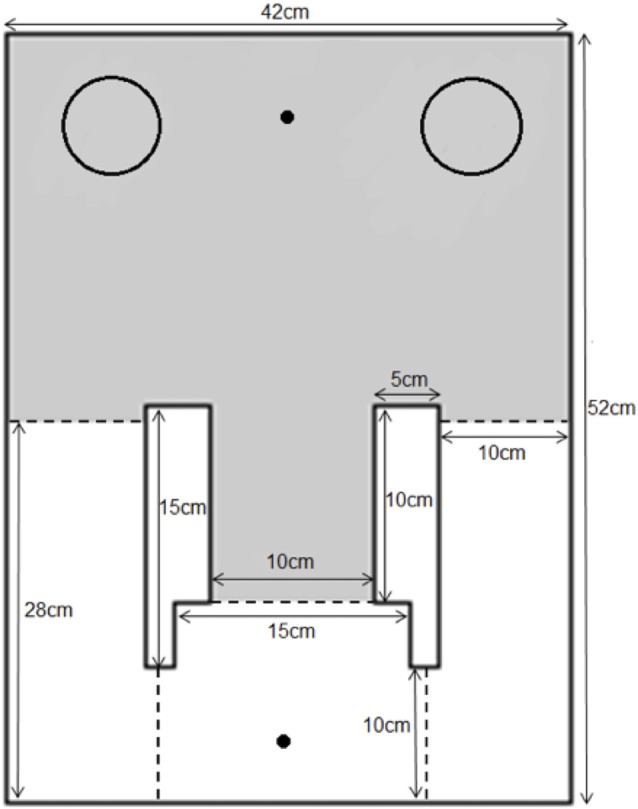
The mouse continual trials apparatus. Mice were placed into the apparatus in the holding area (white area in figure) and doors (dotted lines) were opened to allow the animal through the central arm into the object area (gray area in figure). Objects were placed in the corners of the object area (indicated on figure by circles) and liquid food reward was delivered in both areas to reinforce shuttling between the two areas (food delivered to location marked by dot in figure).

The apparatus used in this experiment was modified from the continual trials spontaneous recognition apparatus for rats (Ameen-Ali et al., 2012; Seel et al., 2017) and adapted in size for use with mice. A rectangular area (50 cm × 42 cm × 20 cm) comprised of a holding area and an object area. The two areas were divided by black guillotine doors of which the width of the outer arm doors measured at 10 cm and the central arm door measured at 15 cm. The animal traveled through the central door to enter the object area and through the two side doors to travel through into the holding area. As with the apparatus used by Ameen-Ali et al., 2012, we believe that the use of different doors to enter and leave the apparatus provide cues to the animal that distinct aspects of the task (e.g., sample phase or test phase) have started and ended. A schematic diagram and an image of the apparatus can be seen in Figure 1. The doors were operated by being lifted from above by the experimenter during the experiment to allow the animal to shuttle from the holding area to object area and vice versa. During the experiment, the objects were placed at the back-left and back-right corner of the object area with a distance of approximately 3 cm from the walls to allow optimum object exploration. Two food wells, one each in the holding and object areas, were located in the middle of the far end walls of the apparatus. This is a modification from the rat apparatus reported by Ameen-Ali et al. (2012), in which food wells were presented next to each object. As the food presented to the animal was not to reward “correct” behavior, but rather to motivate the animal to shuttle between the holding and object areas, we modified the delivery of food reward in this instance to separate it from the objects themselves, minimizing the likelihood that animals associate the food either directly with a particular object or with a particular choice of object. The apparatus was made out of 10 mm opal acrylic and the floors of the apparatus comprised of a gray Lego™ surface. The apparatus was covered by a clear Perspex roof and an overhead camera was fixed at a height of 1.0 m above the apparatus to provide a top-down view of the apparatus. The apparatus was placed in a room illuminated by diffuse lighting originating from a 50 w lightbulb reflected off the walls. White noise was continuously played in the background during the course of the experiment to mask any extraneous noise. The experimenter was present in the room throughout the experiment and visible to the animal when operating the doors, though stood out of sight of the animal when the animal was exploring objects.

### Subjects

Sixteen experimentally naive female (*n* = 8) and male (*n* = 8) C57bl/6j mice (Charles River, UK) and 16 experimentally naïve female (*n* = 8) and male (*n* = 8) TASTPM mice (GlaxoSmithKline, UK), overexpressing the hAPP695swe mutation (TAS10) and the Presenilin-1 M146V mutation and backcrossed with C57Bl/6J mice (Howlett et al., 2004) were used as subjects in this experiment. The animals were housed in groups of up to four in individually ventilated cages under diurnal conditions (12-h light-dark cycle; 07:00–19:00 h). All procedures were conducted in accordance with the requirements of the United Kingdom Animals (Scientific Procedures) Act 1986 and associated guidelines, under the remit of Home Office license number 70/7785. These procedures were also approved by the local animal welfare ethical review board at Durham University. Behavioral testing occurred during the light phases of the day. The animals were food deprived to 90%–95% of their free feeding weight and thus maintained throughout the study. Water was available *ad libitum*. The animals weighed between 20.8 and 39.6 g at the start of testing. The c57 and TASTPM mice were tested at separate time points from one another.

Each strain of mice was split into two groups, one (12 c57s and 8 TASTPM) tested at both 7 and 10 months of age, and therefore experienced with the behavioral testing at 10 months, and one (4 c57s and 8 TASTPM) that were tested only at 10 months of age, at which point they were naïve to the behavioral testing. TASTPM mice experienced high levels of mortality at a relatively young age, as previously reported (Pugh et al., 2007), meaning that numbers of TASTPM mice run in each task at each age point varied. In addition, animals were only included in the analysis if they completed the habituation and training for that task. Numbers of animals for each task at each age are reported with the results. Ages 7 and 10 months of age were chosen for testing the mice based on reported pathology of the TASTPM mice. Aβ deposits are routinely observed from 6 months of age in TASTPM mice, and increase with age (Howlett et al., 2004). Impairments in spontaneous recognition impairments have been observed in TASTPM mice as young as 6 months of age (Howlett et al., 2004). As a result, TASTPM animals tested in the current study at 7 months of age will show pathological changes which will increase in the 10-month-old animals.

### Behavioral Testing

#### Habituation

Before experimental testing, animals were given at least 7 days after arrival to acclimatize to their new environment before any handling. Subsequently, they received 5 min habituation to handling sessions every day for 5 days. Finally, the animals were taken in their home cage with their cage mates to the experimental testing room for 10 min to acclimatize to this environment.

#### Pre-training the Continual Trials Approach

After acclimatizing to the experimental room, animals were pre-trained in the continual trials apparatus in order to become habituated with the environments and learn to shuttle between trials. All stages of pre-training occurred on different days. At stage 1, mice were placed in the apparatus in cage groups to allow free exploration of the maze for 30 min. The side arm doors and central door was removed to allow the animals to freely explore the apparatus without obstruction. Mice were encouraged to explore the apparatus by placement of 0.1 ml (50% vol) sweetened condensed milk (Nestlé); milk was placed at random all over the floors of the apparatus. 1.0 ml sweetened condensed milk solution was allocated to each mouse. Mice progressed to stage 2 pre-training once 80% of milk solution was consumed within the 30-min session. At stage 2, mice were placed into the apparatus on their own to freely explore the apparatus (as in stage 1) for a total of 20 min. 0.1 ml droplets of 50% sweetened condensed milk were placed randomly on the floors of the apparatus totaling 1 ml for each mouse. Animals moved to stage 3 when they had consumed at least 80% of the milk in a 20-min session.

Stage 3 trained mice to shuttle in the apparatus. The animal was initially placed into the holding area which contained a drop of sweetened condensed milk solution. Once the animal consumed the milk, the experimenter opened the central door to allow the mouse to shuttle through to the object area. As soon as the animal entered the object area, the experimenter shut the central door and replenished the food well in the holding area. Once the animal had consumed the food in the object area, the experimenter opened the side arm doors to allow the animal to enter the holding area to retrieve food. After the animal returned to the holding area, the experimenter closed the side arm doors and replenished the food in the object area. This procedure was repeated over a 10-min training session. Animals progressed to the next stage when they were able to immediately shuttle between the holding and object area within 10 s (typically three sessions over three consecutive days). If an animal failed to shuttle from one compartment to the other within 5 min, the session timed out and the animal was removed from the apparatus.

Stage 4 exposed animals to objects in the apparatus. Mice were initially placed in the holding area, the central doors opened to allow the animal to shuttle into the object area where a pair of identical objects was placed at the far corners of the object area at a distance of 3 cm from the walls. After 2 min in the object area, the side arm doors opened so the mice were able to shuttle back into the holding area where the animal would wait for a 1-min inter-trial interval during which the experimenter replaced the objects with a new pair of identical objects. This protocol was repeated until mice were exposed to four pairs of objects. As with stage 3, 0.1 ml droplets of condensed milk were replenished in the holding and object areas each time they were consumed by the mouse. The animals did not re-encounter the objects from stage 4 during the experimental testing.

#### Object Recognition Task (OR)

For each animal a session constituted 16 trials. As with the continual trials approach in rats (Ameen-Ali et al., 2012; Seel et al., 2017) the number of trials is not limited due to potential performance of the animals. We see no evidence that animals’ exploration or engagement with the task is different on trial 16 to trial 1. Rather, 16 trials allow a counterbalanced testing schedule and runs for just under 2 h, after which time we allow the animals to return to their home cage to socialize and return to *ad libitum* water.

A single trial structure was as follows: sample phase, a retention delay, a test phase and an inter-trial interval. The mouse was initially placed in the holding area of the apparatus. To start the session, the central door opened to allow the animal to shuttle through into the object area of the apparatus which contained a pair of identical, trial unique, objects (e.g., object “A”), each located at the back-left and back-right corners of the apparatus. The animals were given 2 min to explore the objects in the object area. At the end of the sample phase, the side doors were opened to allow the animal to return to the holding area for 1 min during which time the objects were changed to prepare for the test phase. At the end of this 1-min period, the central door opened once more and the animal shuttled back into the object area of the apparatus for the test phase during which the animal would be presented with a copy of the familiar object (e.g., “A”) and a novel object (e.g., object “B”) for 2 min. At the end of this period, the side doors opened and the animal was allowed to return to the holding area for a 1-min inter-trial interval. The session ended after 16 such trials had been run consecutively by the animal.

0.1 mL of 50% sweetened condensed milk solution was replenished in both the holding and object area each time after the animal shuttled to the next compartment (as food was placed centrally in each area there was no reinforcement of object choice from this food, rather it acted purely as a motivator to encourage animals to shuttle back and forth for the 16 trials of a session). The novel object was presented on the left for half the trials and on the right for the other half. The identity and location of the novel object, and the order in which objects were presented across trials was counterbalanced as far as possible given the number of mice. The criteria for ending the testing session occurred when the animal failed to shuttle to the next compartment within 3 min of the door opening, or at the end of the prescribed 16 trials. If the animal failed to shuttle within the allotted time frame, the testing session would cease and the animal would be excluded from the data analysis of the experiment.

#### Object Location Task (OL)

As for object recognition (OR) the session began with an animal placed in the holding area and the central door being opened so the animal could shuttle into the test area. In the test area were two trial unique novel objects (e.g., objects “A” and “B”). At the end of 2 min during which the animal was free to explore, the side door was opened to allow the animal to return to the holding area for 1 min, during which time the objects were prepared for the test phase. At the end of this 1-min, the central door opened to allow the animal to shuttle back into the object area which now contained two copies of one of the previously seen objects (e.g., A). Copies of the objects seen in the sample phase were used in order to ensure there were no odor cues from the animals’ previous exploration of the objects. One of the identical objects (e.g., A) was located in the familiar location (i.e., the location filled by the same object in the sample phase) and the other (e.g., A) in novel location (i.e., in the location previously filled by object B in the sample phase). At the end of 2 min exploration time, the side doors were opened once more and the animal shuttled back to the holding area for a 1-min inter-trial interval during which time the objects were prepared for the next trial. This procedure was repeated for 16 trials.

As with the spontaneous OR task, the novel object was presented on the left for half the trials and on the right for the other half. The identity and location of the novel object, and the order in which objects were presented across trials was counterbalanced as far as possible given the number of mice. If animals failed to shuttle between compartments within 3 min, the session would time out, the animal would be removed and would then be excluded from the analysis.

## Results

Mice aged 7 and 10 months were assessed in the continual trials apparatus on both OR and OL memory. When tested at 10 months of age, additional animals, naïve to the apparatus and the experimental protocol, were added to the cohort being tested to examine the effects on performance of previous experience in the apparatus. Comparisons across ages were only made for animals that completed the task at both ages. In all cases animals ran 16 trials of the given task in a single test session and performance was assessed across the whole of the session as well as in blocks of the first and last two trials in each session. Sex of the mice had no significant effect on the results at any point and so all data represents combined performance of males and female mice.

Statistical comparisons against chance were carried out by one-way *t*-tests whilst differences in performance for animals completing the task at both ages were assessed through paired *t*-tests. Independent *t*-tests were used to compare experienced and naïve animals at 10 months of age.

In all cases, memory performance was assessed using a discrimination ratio (D2) calculated as exploration of novel-exploration of familiar/total exploration. This gives a range of performance from 1 (exclusive exploration of the novel object or OL configuration) to −1 (exclusive exploration of the familiar object or OL configuration) with chance at 0 indicating equal exploration of each object. As each animal completed 16 trials within a single test session, D2 was calculated on trial 16 through the cumulative exploration of novel and familiar objects up to trial 16 (rather than averaging the D2s of individual trials). This is in line with similar methods of assessing spontaneous recognition memory using a continual trials approach (Albasser et al., 2010; Ameen-Ali et al., 2012; Seel et al., 2017). Calculating a D2 from the total exploration across the 16 trials (rather than averaging D2s calculated for each trial individually) ensures that the measure of performance is not unduly influenced by individual trials in which very small amounts of object exploration occurred.

**Figure 2:**
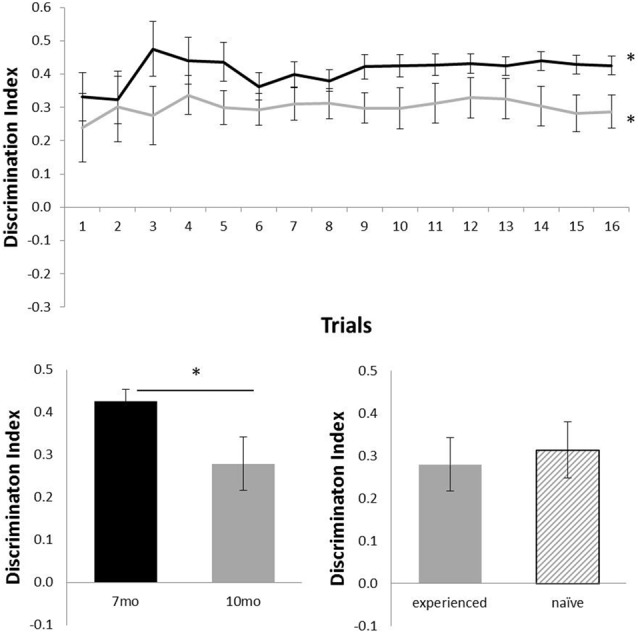
Performance of c57 mice in object recognition (OR) at 7 (black) and 10 (gray) months of age (error bars represent SEM; *represents a significant effect). Top panel is cumulative D2 at each of the 16 trials. Bottom left panel is cumulative D2 for animals that completed testing at both ages. Bottom right panel reflects D2 at 10 months of age for experienced animals (also tested at 7 months) and naïve animals (only tested at 10 months of age).

Figure 2 shows the performance of c57 mice in the OR task (7 months of age, *N* = 12; 10 months of age, *N* = 16). Animals were above chance at both 7 months (*N* = 12; mean = 0.43; *t*_(11)_ = 15.06, *p* < 0.001) and 10 months (*N* = 16; mean = 0.29; *t*_(15)_ = 5.87, *p* < 0.001). At 10 months, experienced (*N* = 12) and naïve (*N* = 4) animals did not differ in performance (*t*_(14)_ < 1). Age had a significant effect on performance: animals that carried out the OR task at both 7 and 10 months of age showed a significant decline in performance from 7 months (mean = 0.43) to 10 months of age (mean = 0.28; *N* = 12; *t*_(11)_ = 2.41, *p* < 0.05).

**Figure 3:**
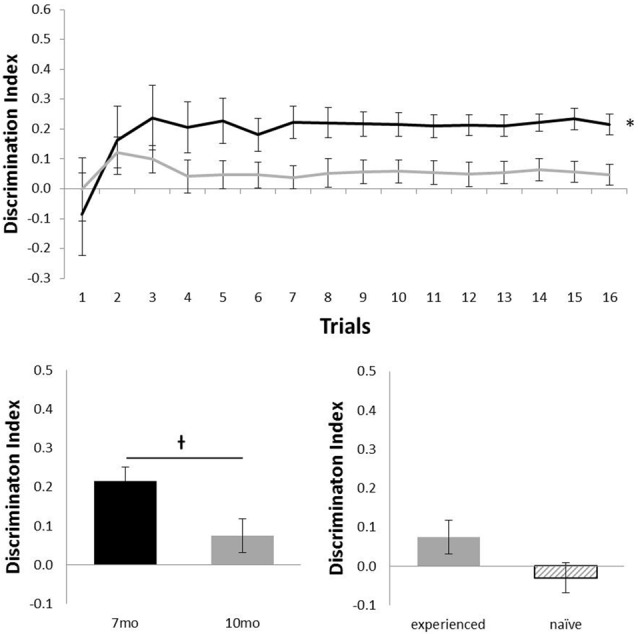
Performance of c57 mice in object-location (OL) at 7 (black) and 10 (gray) months of age (error bars represent SEM; *represents a significant effect, ^†^represents a non-significant trend). Top panel is cumulative D2 at each of the 16 trials. Bottom left panel is cumulative D2 for animals that completed testing at both ages. Bottom right panel reflects D2 at 10 months of age for experienced animals (also tested at 7 months) and naïve animals (only tested at 10 months of age).

Figure 3 shows the performance of c57 mice in the object location (OL) task (7 months, *n* = 12; 10 months, *n* = 16). Animals performed at above chance levels at 7 months (*N* = 12; mean = 0.21; *t*_(11)_ = 6.01, *p* < 0.001), but performance was not above chance at 10 months of age (*N* = 16; mean = 0.05; *t*_(15)_ = 1.37, *p* = 0.189). Although there was no statistical effect of ageing in this task (*N* = 12; *t*_(11)_ = 2.11, *p* = 0.06), the difference in age did show a possible non-significant trend for older animals to perform more poorly. At 10 months of age, experience had no effect on performance (*N*_experienced_ = 12, *N*_naive_ = 4; t_(14)_ = 1.32, *p* = 0.21).

**Figure 4:**
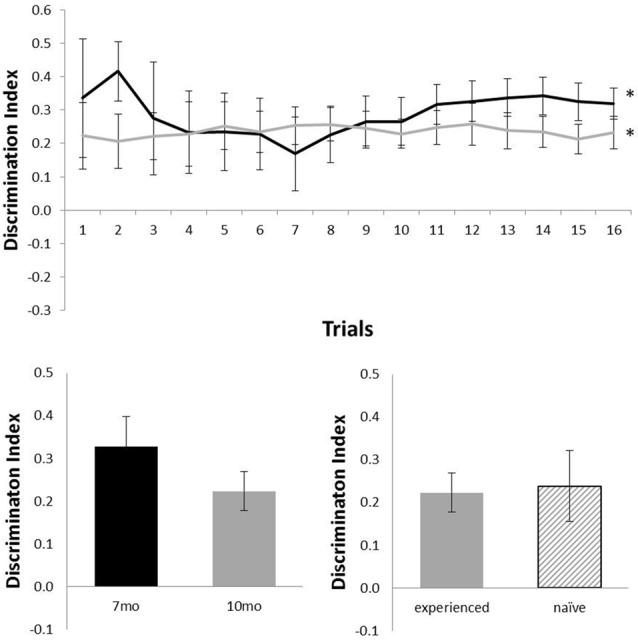
Performance of TASTPM mice in OR at 7 (black) and 10 (gray) months of age (error bars represent SEM; *represents a significant effect). Top panel is cumulative D2 at each of the 16 trials. Bottom left panel is cumulative D2 for animals that completed testing at both ages. Bottom right panel reflects D2 at 10 months of age for experienced animals (also tested at 7 months) and naïve animals (only tested at 10 months of age).

Figure 4 shows performance of TASTPM mice in the OR task (six mice at 7 months of age and nine mice at 10 months of age). As TASTPM animals experience a higher rate of death than c57 mice, not all animals tested at 7 months of age were able to be tested at 10 months of age, and not all animals held to be introduced at 10 months of age were able to be introduced). TASTPM mice performed above chance levels at both 7 months of age (*N* = 6; mean = 0.32; *t*_(5)_ = 6.76, *p* = 0.001) and 10 months of age (*N* = 9; mean = 0.23; *t*_(8)_ = 4.77, *p* = 0.001). The performance of naïve (*N* = 5) and experienced (*N* = 4) animals did not significantly differ at 10 months (*t*_(7)_ < 1). Those TASTPM animals that carried out the OR task at both 7 and 10 months of age showed no significant decline in memory from 7 months (mean = 0.33) to 10 months of age (*N* = 4; mean = 0.22; *t*_(3)_ = 1.39, *p* = 0.26).

**Figure 5:**
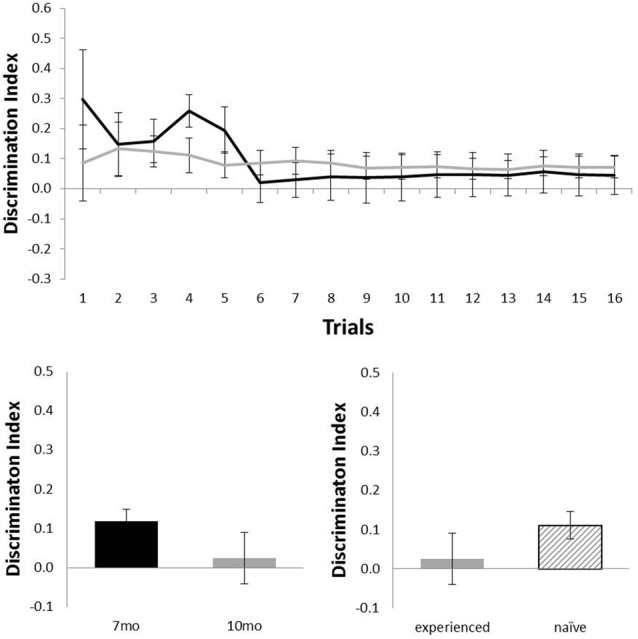
Performance of TASTPM mice in OL at 7 (black) and 10 (gray) months of age (error bars represent SEM). Top panel is cumulative D2 at each of the 16 trials. Bottom left panel is cumulative D2 for animals that completed testing at both ages. Bottom right panel reflects D2 at 10 months of age for experienced animals (also tested at 7 months) and naïve animals (only tested at 10 months of age).

Figure 5 shows performance of the Alzheimer’s Disease model (TASTPM) mice in the OL task (7 months, *n* = 6; 10 months, *n* = 9). TASTPM mice did not perform above chance levels at 7 months of age (*N* = 6; mean = 0.05; *t*_(5)_ < 1) or at 10 months of age (*N* = 9; mean = 0.07; *t*_(8)_ = 1.98, *p* = 0.08), and performance at 10 months of age was not affected by previous experience (*N*_experienced_ = 4, *N*_naive_ = 5; *t*_(7)_ = 1.26, *p* = 0.25). Those TASTPM animals that carried out the OL task at both 7 and 10 months of age showed no significant decline in memory from 7 months (mean = 0.12) to 10 months of age (*N* = 4; mean = 0.02; *t*_(3)_ = 1.57, *p* = 0.22).

**Figure 6:**
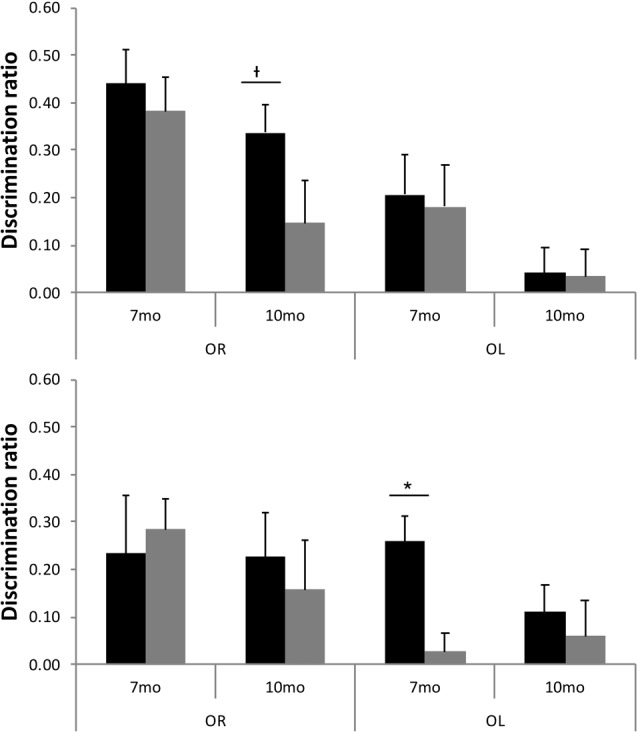
D2 for first four trials (black bars) and final four trials (gray bars) for both OR and OL tasks at 7 and 10 months of age in c57 (top panel) and TASTPM (bottom panel) mice (error bars represent SEM; *represents a significant effect, ^†^represents a non-significant trend).

Although TASTPM animal’s performance on the OL task was not above chance levels, when assessed across all 16 trials, at either 7 or 10 months of age (Figure 5), the performance on early trials appears to be good at 7 months of age. Although the continual trials approach gives improved reliability of the data (through reduced behavioral noise), the approach of multiple trials of similar events one after the other raises the significant possibility of increased interference compared to more typical one trial a day task. Although the objects change in a trial unique manner, the locations used within the arena, the context of the arena and the location of the arena in the room are all constants across trials. As a result, it is possible that continual trials measures of spontaneous recognition memory become more difficult as the animals progressed through the session as encoding events become increasingly difficult to separate from one another. To test whether this was the case for the TASTPM animals in the OL task, we used paired *t*-tests to compare the D2 derived from cumulative exploration data for the first four trials of the session (as a point with hypothesized low interference from previous trials) with the last four trials of the session for each animal (as a point with hypothesized high levels of interference from previous trials). These data are shown in Figure 6. For TASTPM animals at 7 months of age there is a significant difference between performance on the first four trials (mean = 0.26) and last four trials (mean = 0.03; *t*_(5)_ = 3.19, *p* = 0.02). In this case performance on the first four trials is itself also significantly above chance (*t*_(5)_ = 4.77, *p* = 0.005) and performance on the last four trials is not (*t*_(5)_ < 1). This is the only example in which overall performance was seen to be significantly different between the first and last four trials in this way. In c57 mice there was a difference between first and last trials in the OR task at 10 months of age which approached significance (t_(15)_ = 1.91, *p* = 0.08). In this condition performance was once again above chance for the first four trials (mean = 0.34; *t*_(15)_ = 5.87, *p* < 0.001) but not different to chance for the final four trials (mean = 0.15, *t*_(15)_ = 1.62, *p* = 0.13). In all other comparisons there was no difference between performance in the first and last four trials of a task (in all cases *t* < 1).

Table 1 presents exploration times for each group on each task. As expected, the more subtle novelty in the OL task (where the object and the location on their own are both familiar, only the combination of object and location is novel) is reflected by lower exploration times. However, in each case exploration times over the 16 trials are high. As the D2s used in the analysis are generated from these cumulative exploration times (not from averaging individual D2s from each session) this means no D2 is arrived at as the ratio of two small amounts of exploration. As a result, we can be confident that significant differences in D2 arise through real differences in exploration of the novel and familiar objects or OLs, and are not the result of a D2 over estimating the performance by comparing two low levels of exploration. We also note that for the c57 animals, exploration times at 7 months of age were lower than at 10 months of age. This was not true of the TASTPM animals. It is unclear from the current study whether these differences reflect true behavioral differences between the strains or whether they are the result of chance variation given the relatively large individual differences in exploration rates across animals.

**Table 1:** Group average cumulative exploration times on the final trial for novel, familiar and both objects in seconds.

Strain	Age	Task	Number of animals in group	Mean exploration of novel object in seconds	Mean exploration of familiar object in seconds	Mean total exploration of objects in seconds
C57	7 months	OR	12	239.4	94.3	333.7
		OL	12	157.6	103.1	260.7
	10 months	OR	16	331.9	179.8	511.7
		OL	16	200.7	179.0	379.7
TASTPM	7 months	OR	6	348.0	172.9	520.9
		OL	6	201.0	174.9	375.9
	10 months	OR	9	309.0	193.8	502.8
		OL	9	213.8	188.2	402.0

*Data is presented for each task (OR, object recognition and OL, object-location recognition) at both 7 and 10 months of age for both c57 and TASTPM mice. In each case the number of animals in the group is also presented*.

## Discussion

We have demonstrated the effectiveness of a new, continual trial, approach to spontaneous recognition tasks in mice. Although spontaneous recognition tasks are widely used, they are behaviorally noisy tasks that require significant numbers of animals to produce sufficient statistical power and are sensitive to environmental factors which can mask or mimic memory impairments (Ennaceur, 2010). The effects of large scale behavioral noise mean that results from these tasks can be sometimes unreliable, which is a significant concern given the focus on failure to translate the findings from memory experiments in animal models of disease into treatments for memory problems (e.g., Cummings et al., 2014).

The continual trials approach produces a significant reduction in the number of rats needed to maintain statistical significance (Ameen-Ali et al., 2012, 2015), and this element of animal reduction is an important element of this new approach. Here we demonstrate that this same reduction is achieved in mice. A power analysis derived from our c57 7-month old mice show for OR a power of 0.987 and a minimum sample size of three. For the same mice in OL a power analysis shows a power of 0.977 and a minimum sample size of six. These data can be compared directly to previous data from mice in our own studies using one trial a day approaches for OR and OL (Davis et al., 2013). In the previous study 12 mice were used and the power analysis from those animals demonstrated for OR a power of 0.95 and a minimum sample size of eight and for OL a power of 0.95 and a minimum sample size of nine. Therefore, our current continual trials approach produces the same (if not greater) statistical power from fewer animals in both the OR and OL tasks.

However, reduction of behavioral noise through this improved method brings other benefits. For example, here we show that c57 mice show an age dependent decline in OR memory between 7 and 10 months of age, but that in both cases performance is above chance (Figure 2). These results support the notion that age affects OR memory (Dunnett et al., 1988). In standard one-trial a day versions of the spontaneous OR task, however, high levels of behavioral variability means that whilst impairments can often be seen by performance above chance in one group and not in another, statistical differences between groups where both groups are above chance are rarely reported (Ameen-Ali et al., 2015). Being able to observe the effects of ageing on performance of the task whilst performance remains above chance at all ages is primarily a result of the reduced behavioral noise from the use of the continual trials approach described here. As a result, we believe that the continual trials approach to spontaneous OR allows much finer grade distinctions between OR memory in animals with different manipulations. In contrast, OL memory at the same ages shows above chance performance in younger animals, but 10-month-old animals’ performance was not above chance (Figure 3). Although both tasks here show differences in performance as a result of age, only the object location task shows animals cannot perform the task above chance at 10 months of age.

This improved sensitivity of this continual trials approach allows us to also gain a better understanding of disease models tested on these tasks of recognition memory. Here the TASTPM mouse was tested at both 7 and 10 months of age and above average performance was seen in TASTPM mice on the OR task at both ages (Figure 4), but in contrast these same animals did not show above chance performance on the OL recognition task at either age (Figure 5). The sensitivity to memory decline shown in c57 mice allows us to be confident that this pattern of results reflects a genuine difference in performance of object and OL recognition in these TASTPM mice. TASTPM pathology includes neuronal loss in the hippocampus (Howlett et al., 2008), meaning this dissociation in function is expected in line with the view that OR memory is independent of normal hippocampal function (Aggleton and Brown, 1999) but is in contrast to others where the hippocampus is considered crucial in both these tasks (e.g., Clark et al., 2000).

The continual trials approach also gives an insight into performance on these recognition memory tasks beyond whether the animals are above chance or not. The nature of consecutive trials with many overlapping features means that proactive interference is likely to occur (Albasser et al., 2010) and can give further insight into memory. Here we see two contrasting ways in which such proactive interference can give insight into the effects of ageing on behavior and disease, neither of which would have been detectable by a typical one-trial a day approach to spontaneous recognition memory. In the TASTPM animals, 7-month-old animals appear to fail the OL recognition memory task. Performance at the end of the 16 trials is not above chance (Figure 5). However, performance on the first four trials is above chance, whilst performance on the final four trials is not above chance, and there is a significant difference in performance between these distinct blocks of trials (Figure 6). This pattern of results clearly shows that normal performance on OL recognition is not beyond the ability of 7-month-old TASTPM mice. Rather, their ability to perform well in the face of significant proactive interference is impaired. In these mice, then, a sensitivity to proactive interference is seen in animals with impaired performance but independent of task and independent of overall performance on the task. These can also be contrasted with animals that show strong reliable performance on a task and no sign of proactive interference (e.g., c57 mice at 7 months of age on OR) and mice that show severe impairment in overall performance and no sign of normal performance in early trials in which proactive interference is minimal (e.g., c57 mice at 10 months of age on OL recognition). These differences mean that alongside other approaches to recognition memory tasks (Albasser et al., 2010) comparison of performance across groups and across tasks is no longer just limited to overall performance, but on the pattern of performance over consecutive trials.

As a result of the improved reliability and the ability to explore patterns of impairment as well as overall performance, the continual trials approach allows us here to answer critical questions about recognition memory in TASTPM mice. To our knowledge no one has previously reported performance of TASTPM animals on the OL task. However, TASTPM mice have been shown to have impairments in object memory (Howlett et al., 2004) but in a way that is not always reliable across studies (Scullion et al., 2011). Where these studies of OR have relied on one-trial a day approaches this lack of reliability is likely a result of the large amount of behavioral noise included in this approach. For example, our findings that TASTPM mice at 7 months old can perform OL memory in the first trials of the task, but not show an overall performance above chance is a subtlety that cannot be detected in these earlier studies. In contrast these findings may have manifested themselves either as preserved or impaired performance on one-trial a day approaches, leading to conflicting results in the literature. The benefit of the continual trials approach to spontaneous recognition memory then is not only the reduction in animal numbers, but the overall reduction in number of studies needed as a result of improved reliability and sensitivity from these tasks.

## Author Contributions

MC designed, ran and analyzed the experiments and contributed to the manuscript. AE, ME and DS designed experiments and contributed to the manuscript. MS and JW provided materials and animals for the experiments and contributed to the design.

## Conflict of Interest Statement

JW and MS were employed by GSK. The remaining authors declare that the research was conducted in the absence of any commercial or financial relationships that could be construed as a potential conflict of interest.
